# Clinical features, epidemiology, autoantibody status, HLA haplotypes and genetic mechanisms of type 1 diabetes mellitus among children in Qatar

**DOI:** 10.1038/s41598-021-98460-4

**Published:** 2021-09-23

**Authors:** Basma Haris, Ikhlak Ahmed, Najeeb Syed, Hakeem Almabrazi, Saras Saraswathi, Sara Al-Khawaga, Amira Saeed, Shihab Mundekkadan, Idris Mohammed, Sanaa Sharari, Iman Hawari, Noor Hamed, Houda Afyouni, Tasneem Abdel-Karim, Shayma Mohammed, Amel Khalifa, Maryam Al-Maadheed, Mahmoud Zyoud, Ahmed Shamekh, Ahmed Elawwa, Mohammed Y. Karim, Fawziya Al-Khalaf, Zohreh Tatari-Calderone, Goran Petrovski, Khalid Hussain

**Affiliations:** 1 Division of Endocrinology, Department of Paediatric Medicine, Sidra Medicine, Weill Cornell Medicine-Qatar, Al Luqta Street, Education City North Campus, PO Box 26999, Doha, Qatar; 2 Translational Research, Sidra Medicine, Doha, Qatar

**Keywords:** Type 1 diabetes, Haplotypes

## Abstract

To describe the clinical features, epidemiology, autoantibody status, HLA haplotypes and genetic mechanisms of type 1 diabetes mellitus (T1DM). Patients (0–18 years) with diabetes were recruited. Clinical data was collected, autoantibodies and c-peptide were measured. Whole Genome Sequencing was performed. Genomic data analysis was compared with the known genes linked with T1DM and HLA alleles were studied. 1096 patients had one or more antibody positivity. The incidence of T1DM in 2020 was 38.05 per 100,000 children and prevalence was 249.73. GADA was the most common autoantibody followed by IAA. Variants in *GSTCD, SKAP2, SLC9B1, BANK1* were most prevalent. An association of HLA haplotypes DQA1*03:01:01G (OR = 2.46, *p* value = 0.011) and DQB1*03:02:01G (OR = 2.43, *p* value = 0.022) was identified. The incidence of T1DM in Qatar is the fourth highest in the world, IA2 autoantibody was the most specific with some patients only having ZnT8 or IA2 autoantibodies thus underlining the necessity of profiling all 4 autoantibodies. The genes associated with T1DM in the Arab population were different from those that are common in the Caucasian population. HLA-DQ was enriched in the Qatari patients suggesting that it can be considered a major risk factor at an early age.

## Introduction

Type 1 diabetes mellitus is the most common form of diabetes observed in children. It is a chronic multifactorial disease with a strong genetic component, which, through interactions with specific environmental factors, triggers disease onset. Type 1 diabetes mellitus usually presents itselft in early to mid childhood as a defect in insulin production through the autoimmune destruction of pancreatic beta-cells^1^. There are two forms of type 1 diabetes mellitus (1A autoimmune and 1B idiopathic). In the autoimmune type there is antibody mediated beta-cell destruction resulting in metabolic abnormalities which is manifested as impaired glucose tolerance first and then progresses to symptomatic hyperglycaemia. Approximately 50% of the familial clustering of genes, which increase the susceptibility risk of inheriting type 1 diabetes mellitus, are located within or in the Human Leucocyte Antigen (HLA) complex on chromosome 6^2^.

The performance of high-density Genome Wide Association Studies (GWAS) enabled by the advent of high-throughput single nucleotide polymorphism (SNP) genotyping array technologies, many additional type 1 diabetes mellitus susceptibility loci and genes have now been discovered^3^. Recent meta-analyses of multiple datasets from independent investigators have brought the total of genes implicated in type 1 diabetes mellitus to nearly 60^4^.

Autoimmunity in human type 1 diabetes mellitus relies on the detection of insulitis, islet cell autoantibodies and activated beta-cell-specific T lymphocytes. The first autoantibodies described in association with the development of type 1 diabetes mellitus were islet cell autoantibodies (ICA)^5^. Subsequently, autoantibodies to insulin (IAA), glutamic acid decarboxylase (GADA), protein tyrosine phosphatase (IA2 or ICA512) and Zinc (ZnT8) have all been defined. The appearance of a beta-cell autoantibody, the first of which is usually against either insulin or GADA (sometimes two autoantibodies appear together) occurs very early on in childhood^6,7^. The first islet autoantibody is rarely directed against islet antigen-2, whereas the likelihood of it being against ZnT8A remains to be determined^7^. These four biomarkers of type 1 diabetes mellitus are strong predictors of disease because the development of a first autoantibody and subsequent development of second, third, and often a fourth one has been reported to result in the clinical onset of type 1 diabetes mellitus in 100% of patients when followed up for 20 years^5^.

The type 1 diabetes mellitus autoantibody and genomic data in the middle-east region is limited. Some studies from Saudi Arabia, UAE, Bahrain have reported the autoantibody status in their cohorts and most of them agree that GADA is the most common autoantibody in Arab ethnicity as well^8–11^.

Association of HLA alleles with susceptibility to type 1 diabetes mellitus, has been the subject of intense investigations during the past decades and have resulted in the description of HLA alleles- DRB1*04, DQA1:03:01 and DQB1:03:01 as a strong indicator of the disease^12,13^. However most of these studies were conducted in individuals of European descent. In some ethnic backgrounds, HLA-A and -B alleles have been shown to affect type 1 diabetes mellitus susceptibility independently from class II molecules, in particular in younger-onset patients^14–16^.

In this study we report on the comprehensive autoantibody profile of every child with type 1 diabetes mellitus in the state of Qatar as well as the HLA status and the genomic data. Previous studies from Qatar reported the prevalence of GADA and IA-2A was 75.5% and 53.4% respectively in a type 1 diabetes mellitus cohort and a higher occurrence of IA-2A autoantibody in familial type 1 diabetes mellitus^17,18^. However there are no reported prospective studies from Qatar that studied all prevalent autoantibodies, HLA status and genomics of type 1 diabetes mellitus.

## Methods

### Ethical compliance

This study was approved by the Institutional Review Board (IRB) for the protection of human subjects in Sidra Medicine, Qatar (IRB reference number 1702007592). Informed consent and assent was taken from patients and legal guardians as required. All experiments were performed in accordance with relevant guidelines and regulations.

### Patient recruitment

In this prospective study every child with diabetes (aged 0–18 years), attending the diabetes clinics, or admitted as an inpatient in Sidra Medicine (which is the only paediatric diabetes center in Qatar) from 2018–2020 were included in the study. Clinical details about birth history, gestational age, ethnicity, age of onset of diabetes, family history, BMI, weight, signs of insulin resistance (acanthosis nigricans) and other system involvement were collected from hospital records and documented. Peripheral blood samples were collected for complete antibody profiling for-all 4 autoantibodies namely Glutamic Acid Decarboxylase 65 (GADA), Insulin Autoantibody (IAA), Islet Antigen-2 Auto Antibody (IA-2A) and Zinc Transporter 8 (ZnT8A) were measured and values recorded, C-peptide, Tissue transglutaminase (TTG) and Thyroid Peroxidase (TPO) autoantibodies were also measured. Samples were also collected for DNA extraction. Patients with diabetes and atleast 1 autoantibody positivity were classified as type 1 diabetes mellitus, based on ADA guidelines. Patients initially thought to be type 1 diabetes mellitus with all autoantibody negative were reclassified as type 1b diabetes^19^. 81 control patients without diabetes were also recruited and autoantibody testing done. We included as controls children (0–18 yrs) attending the clinic for conditions other than diabetes. The population statistics in 2020 for children living in Qatar was obtained from the Planning and Statistics Authority in Qatar.The epidemiology analysis is performed using epiR package (version 2.0.19) from R Statistics software (version 3.6.3) was used for the prevalence calculations^20^.

### Antibody assay methodology

Complete antibody profiling of every child with diabetes was performed. This was done at the time of recruitment into the study for all known type 1 diabetes mellitus patients. Newly diagnosed cases were tested at the time of diagnosis before starting insulin treatment.

GADA-Radioimmunoassay performed. (125) I-labeled recombinant human glutamic acid decarboxylase is incubated with the patient's diluted serum. Antihuman IgG and IgM are then added to form an immunoprecipitate. After washing the precipitated immune complexes, specific antibodies are detected by counting gamma-emission from the pellet's bound (125)I-GAD65^21^.

Insulin autoantibody-Radioimmunoassay performed. (125) I-labeled recombinant human insulin is added to the test serum, if antibody is present, it forms a soluble complex with the labeled insulin. Subsequent addition of goat antihuman IgG and IgM precipitates the complex. The amount of radioactivity in the precipitate is proportional to the level of antibody in the serum^22^.

IA-2 autoantibody- Radioimmunoassay performed. (125) I-labeled recombinant human IA-2 is added to the test serum, if antibody is present, it forms a soluble complex with the (125) I-labeled IA-2. Subsequent addition of goat antihuman IgG and IgM precipitates the complex. The amount of radioactivity in the precipitate is proportional to the level of antibody in the serum^23^.

Zinc Transporter 8 (ZnT8) autoantibody- Enzyme immunoassay. Zinc Transporter 8 (ZnT8) antibodies are principally directed against the C terminal domain of ZnT8. The ZnT8 autoantibody ELISA is based on the bridging principle that employs the ability of divalent ZnT8 autoantibodies to bind to ZnT8 coated on to the plate well with one arm, and to liquid ZnT8-biotin with the other arm. Calibrators or undiluted serum samples in duplicate are added to ZnT- coated plate wells and incubated overnight. ZnT8-biotin is added to each well and plate. After another incubation, aspiration, and wash, streptavidin-peroxidase is added to each well. Another incubation, aspiration, and wash are performed and peroxidase substrate is added. After a final incubation, 0.5 mol/L H2S04 stop solution is added to each well. Absorbance is measured at 450 nm, blanked against wells containing peroxidase substrate and H2S04 only^24^.

### Genetic testing methodology

DNA samples were extracted from peripheral blood specimen of all individuals recruited into the study including patients and parents in Sidra Medicine, Qatar. Whole Exome Sequencing was used on a set of control subjects and selected patient samples on the basis of autoantibody status, family history of DM and associated clinical features. The 74 patients and 108 control subjects were sequenced on Illumina HiSeq platform using a 150-base paired-end single-index-read format. Reads in FASTQ files were then mapped to the NCBI human reference genome GRGh37/hg19 using Burrows–Wheeler Aligner (BWA-MEM) version 0.7.8. All subjects underwent variant calling using GATK(v3.6) and annotation was performed using SNPEff^25^. Variants file was normalized and decomposed using vt^26^. Additionally, vcfanno^27^ was used to annotate VCF file with extensive available data resources like gnomad, exomes.r2.0.2, gnomad.genomes.r2.0.2.sites, 1 K genome, Exac etc. Genomic variants belonging to 49 genes already known to be implicated in type 1 diabetes mellitus (see Supplementary Table 1) were extracted for patient samples from the multisample VCF file. These variants were further filtered for non-exonic regions using exome bed (Exome-Agilent_V6) and only variants belonging to exome regions were retained for downstream analysis. We looked for the non-synonymous variants which are present in more than 5% in our patient cohort and absent or present in frequency less than 0.1% in public data bases. These variants were considered to be present in a significantly higher frequency in our disease cohort.

### Extraction of HLA data from WGS

We used Population Reference Graph (HLA-PRG)^28^ to identify HLA alleles from the whole exome sequencing of 182 samples comprising of 74 type 1 diabetes mellitus patients and 108 normal controls.

The accuracy of HLA genotypes was assessed on a family-based approach, using 18 full trios (both parents and at least one offspring), and 4 families with only one single founder.

For disease association analysis, the patient and control cohorts were divided into two groups based on the ethnicity of the sample. The association of HLA alleles with type 1 diabetes mellitus phenotype in each group was tested using fisher’s exact test in R and the enrichment of the alleles in cases or controls was assessed using odds-ratio at a p value cut-off of 0.05.

## Results

A total of 1096 patients with diabetes with at least one autoantibody positivity were classified as type 1 diabetes mellitus. The incidence of type 1 diabetes mellitus in 2020 was 38.05 per 100,000 children(0–18 years) with a confidence interval (CI) of 32.5–44.28. A prevalence value of 386.23 per 100,000 children (0.39%) with a CI of 354.93–419.55 was recorded in Qatari children while in non-Qatari children it was 182.57 per 1000,000 children(0.18%) with CI of 167.46–198.67.

### Clinical features

**Figure 1:**
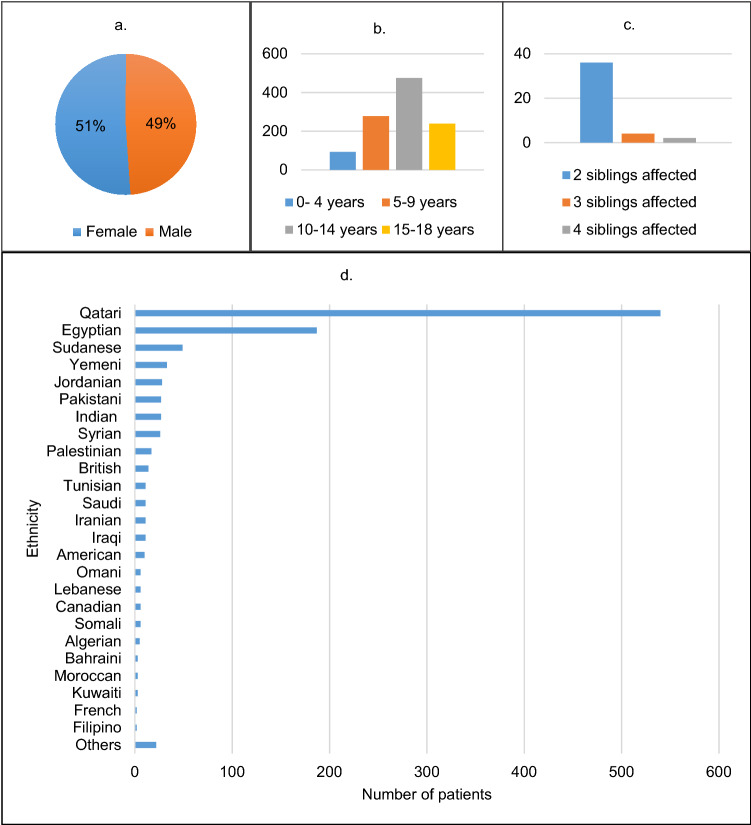
Summary of clinical features of type 1 diabetes mellitus patients. (**a**) Gender distribution of patients. (**b**) Age distribution of patients. (**c**) Number of families with multiple siblings with type 1 diabetes mellitus. (**d**) Ethnicity of type 1 diabetes mellitus patients.

The clinical history of every patient was recorded. Figure 1 summarises some of the clinical features observed and ethnicities of patients with type 1 diabetes mellitus in our cohort. Diabetic ketoacidosis was the most common acute presentation and the onset of diabetes was most common in 5–9 years age groups and uncommon in 15–18 years age group. There was no significant difference noted between male and female affected patients. The blood glucose level at diagnosis for most of our patients was between 300–600 mg/dL. The mean c-peptide level in our cohort was 0.43 ng/mL.

42 families with multiple siblings affected were identified of which 36 families had 2 siblings affected, 4 had 3 siblings affected and 2 families had 4 siblings affected. Table 1 shows associated clinical features observed in some patients of the type 1 diabetes mellitus cohort.

**Table 1:** Associated condition observed in some patients of the type 1 diabetes mellitus cohort.

Associated condition	Number of patients
Celiac disease (TTG positive)	51
Thyroid disease (TPO positive)	215
Short stature	12
Seizure disorder	2
Autism or developmental delay	4
Polycystic kidney disease	1
End-stage chronic kidney disease	1
Myasthenia gravis	1
Hyperammonemia	1
Occulocutaneous albinism	1
Precocious puberty	1
Adrenocortical insufficiency	1

### Antibody assay

**Figure 2:**
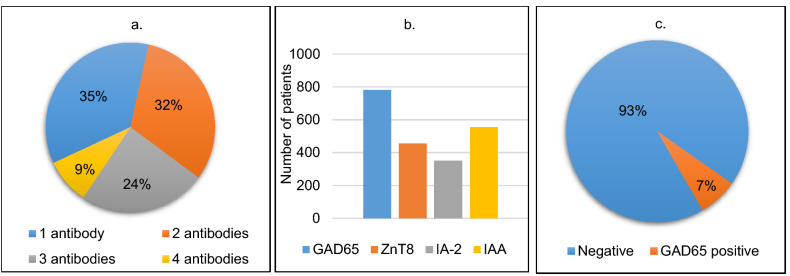
Summary of autoantibody assay on type 1 diabetes mellitus patients. (**a**) Number of type 1 diabetes mellitus patients with 4,3,2 and 1 autoantibody positive. (**b**) Type 1 diabetes mellitus patients with GAD, IA2, IAA and ZnT8 positive. (**c**) Autoantibody assay results in control subjects.

The most common antibody noted in our cohort was GADA closely followed by IAA present in 59.7% and 42.44% of patients respectively. In 6.87% of patients all 4 autoantibodies were positive while 28.17% had only 1 antibody positive. Figure 2a and b summarises the observations from antibody assay in terms of number and type of positive autoantibodies and the proportion of different combinations in our cohort. Antibody assay was also performed in the non-diabetic controls (Fig. 2c).

### Genetic analysis

The variants from 49 known type 1 diabetes mellitus associated genes present at a frequency of > 5% in the patient cohort and < 1% in the gnomad database considered to be enriched in patients and potentially causal. 6 variants in 4 genes-*GSTCD, SKAP2, SLC9B1, BANK1* were found in atleast 5% of the cohort (see Table 2 for full list). Other variants in relevant genes thought to be associated with type 1 diabetes mellitus which are rare in the general population were also considered.

**Table 2:** Variants observed in genes known to be associated with type 1 diabetes mellitus present in our cohort.

Gene	AF	Variant	Effect
GSTCD	0.352	c.980-4delT	splice_region_variant&intron_variant
SKAP2	0.25	c.200-6dupT	splice_region_variant&intron_variant
SLC9B1	0.149	c.1318A > T	stop_gained
GSTCD	0.118	c.980-5_980-4delTT	splice_region_variant&intron_variant
GSTCD	0.083	c.980-9_980-4delTTTTTT	splice_region_variant&intron_variant
BANK1	0.05	c.1209C > A	splice_region_variant
**Variants in < 5% of cohort**			
PTPN22	< 0.05	c.1742A > G	missense_variant
IL18RAP	< 0.05	c.404A > G	missense_variant
IL18RAP	< 0.05	c.1205 T > C	missense_variant
IFIH1	< 0.05	c.1793G > A	missense_variant
STAT4	< 0.05	c.1178G > C	missense_variant
DDIT4L	< 0.05	c.34C > T	missense_variant
TET2	< 0.05	c.1852 T > C	missense_variant
PPA2	< 0.05	c.671C > T	missense_variant
ARHGEF38	< 0.05	c.795C > A	stop_gained
ARHGEF38	< 0.05	c.1772G > A	missense_variant
ARHGEF38	< 0.05	c.1910C > T	missense_variant
SLC9B1	< 0.05	c.1391C > A	missense_variant
ARHGEF38	< 0.05	c.2005G > A	missense_variant
NPNT	< 0.05	c.1114A > G	missense_variant
TNFAIP3	< 0.05	c.2090G > A	missense_variant
COBL	< 0.05	c.3844C > T	missense_variant
IKZF1	< 0.05	c.413C > T	missense_variant
NPNT	< 0.05	c.1302G > T	missense_variant
COBL	< 0.05	c.2507C > T	missense_variant
GLIS3	< 0.05	c.2258A > G	missense_variant
GLIS3	< 0.05	c.706T > G	missense_variant
GLIS3	< 0.05	c.596 + 6A > C	splice_region_variant

### HLA analysis

Our cohort consisted of 182 samples with 74 cases and 108 controls. Among the cases 40 were Qatari ethnicity and 34 non Qatari ethnicity whereas among the controls 39 were Qatari and 69 were non Qatari. A total of 231 alleles were observed for 13 genes with a relatively higher diversity of alleles in HLA-A, HLA-B, HLA-C, HLA-DRB1, HLA-DPB1, HLA-DPA1 and HLA-DQB1 genes (Table 3).

**Table 3:** Distribution of observed alleles across HLA- genes.

HLA-gene	No. of observed alleles	Allele with max freq (cases)	Max allele freq (cases) (%)	Allele with max freq (controls)	Max allele freq (controls) (%)
A	35	A*24:02:01G	20	A*02:01:01G	24
B	51	B*50:01:01G	14	B*51:01:01G	11
C	27	C*06:02:01G	20	C*06:02:01G	17
E	3	E*01:01:01G	58	E*01:01:01G	66
F	2	F*01:01:01G	99	F*01:01:01G	100
G	12	G*01:01:01G	37	G*01:01:01G	39
DPA1	14	DPA1*01:03:01G	78	DPA1*01:03:01G	74
DPB1	20	DPB1*04:01:01G	42	DPB1*04:01:01G	31
DQA1	9	DQA1*05:01:01G	44	DQA1*05:01:01G	33
DQB1	13	DQB1*02:01:01G	53	DQB1*02:01:01G	39
DRB1	37	DRB1*03:01:01G	41	DRB1*03:01:01G	22
DRB3	5	DRB3*02:02:01G	68	DRB3*02:02:01G	53
DRB4	3	DRB4*03:01N	59	DRB4*03:01N	68

Alleles were observed for each gene and the allele(s) with maximum frequency in cases and controls for each gene.

Significant association was detected in Qatari patients with HLA class II, while no significant type 1 diabetes mellitus associations were observed for class I HLA-genes,whereas alleles in both class I and class II genes showed association in non-Qatari samples. There is an enrichment of HLA loci within Qatari patients and controls and indicates an association of -DQA1*03:01:01G (OR = 2.46, *p* value = 0.011) and -DQB1*03:02:01G (OR = 2.43, *p* value = 0.022) with type 1 diabetes mellitus in Qatar (see Supplementary Table 2). Where as, HLA -DQA1*01:02:01G (OR = 0.17, *p* value = 3.4E-03) and -DQB1*06:02:01G (OR = 0, *p* value = 0.014) are enriched in controls and thus potentially protective for the disease in Qatar.

In non-Qatari patients, HLA -DRB1*03:01:01G (OR = 2.51, *p* value = 0.003), was the most significant allele associated with type 1 diabetes mellitus. Four other alleles belonging to class I genes also showed significant associations with the disease (please see Supplementary Table 3). HLA -DRB3*01:01:02G (OR = 0.43, *p* value = 0.014), and -DQB1*03:01:01G (OR = 0.09, *p* value = 0.0019) alleles were most prevalent in controls.

We also observed disparity in the risk imposed by different alleles of the HLA-DQB1*03 gene in Qatari and non-Qatari patients. While HLA -DQB1*03:02:01G is a high frequency allele and significantly enriched in type 1 diabetes mellitus, in Qatar, -DQB1*03:01:01G shows a high frequency in the control group in non-Qatari population.

## Discussion

In this first prospective study we identified a total of 1096 patients aged 0–18 years with type 1 diabetes mellitus. Type 1 diabetes mellitus is the most common type of DM in children with newly diagnosed cases estimated to be 98,200 children under 15 years in the world annually^29^. Our epidemiology data shows that Qatar has the fourth highest incidence of T1DM in the world. The incidence of type 1 diabetes mellitus is highest in Finland and Sweden and is also increasing at an alarming pace in the MENA region as well^30^. No similar nationwide study has been conducted in Qatar or in the MENA region to date and thus our study provides a unique insight into understanding the mechanisms of type 1 diabetes mellitus in Qatar. We can also report that the prevalence of type 1 diabetes mellitus in patients of Qatari ethnicity in our cohort is almost double that of non-Qatari ethnicity. This suggests a unique genetic component conferring increased risk to the Qatari population that goes beyond environmental factors.

A study conducted in Jeddah, Saudi Arabia reported a prevalence of GADA positivity in 65% of their cohort whereas IA-2A was positive in 27%. They also reported that autoantibody positivity is more common if the age of onset of diabetes is at a younger age^8^. A study from Sudan reports 91.2% of children with type 1 diabetes mellitus have developed one or more autoantibodies at onset while 8.2% were seronegative^9^. Another study from eastern province of United Arab Emirates reported 88% had at least 1 autoantibody positive and 13% had 3 autoantibodies positive in their cohort of 61 children with type 1 diabetes mellitus. The most common autoantibodies were GADA and IAA^10^. A study from Bahrain reported the autoantibody status of a selected cohort of 52 newly diagnosed patients with type 1 diabetes mellitus and found GADA, IA-2A and IAA, positive in 71%, 28.9%, and 30.8% respectively of their cohort^11^.

Our study was able to establish GADA and IAA as the most common autoantibodies in the Qatari population being present in 59.7% and 42.44% of all affected children. While GADA is one of the most sensitive test for a diagnosis of type 1 diabetes mellitus^31^ it is not very specific since it was found in some of the controls as well. IA-2 was present in the least number of patients and completely absent in all controls thus suggesting it is more specific to diagnose type 1 diabetes mellitus than the other autoantibodies. 34 patients had only ZnT8 or IA2 autoantibody positivity thus providing strong evidence that testing for GADA and IAA autoantibodies alone is not sufficient, and full antibody profiling is warranted in all patients with diabetes.

Type 1 diabetes mellitus is a disease with a very strong genetic component to its etiology. This is supported by the fact that children born to a family with an affected member have a 5% risk of developing type 1 diabetes mellitus comparted to 0.3% risk in an individual with no affected family members^32^. Previous GWAS studies in European population have identified about 49 genomic loci that are associated with likelihood to develop type 1 diabetes mellitus^33^_._ Apart from the HLA complex, which remains by far the strongest predictor of type 1 diabetes mellitus, the most important loci conferring susceptibility are located in the *INS, PTPN22, IL2RA, SH2B3* genes. The risk for IAA as first autoantibody was associated with *INS, SH2B3, ERBB3, RGS1* and *PTPN22*. GAD as first antibody was associated with *CCR7, SH2B3, TNFAIP3* and *CD226*^34^. However all of these available data is based on Caucasian ethnicity and no such data is available for Arab population. We found multiple genomic variants in *GSTCD, SKAP2, SLC9B1* and *BANK1* genes to be present at a relatively higher frequency in our patient cohort indicating a causal connection between these variants and the incidence of type 1 diabetes mellitus in Qatar. On the other hand, variants in genes *PTPN22, IL18RAP, STAT4,NPNT, GLIS3* known to be associated with type 1 diabetes mellitus from GWAS studies^33^ occur at a relatively lower frequency in our patient cohort. However one limitation of our study was the low number of samples which prevented us from looking for new genes associated with type 1 diabetes mellitus in the Arab population.

The etiology of type 1 diabetes mellitus is multifactorial and the HLA complex is reported to account for about 50% of genetic susceptibility to the development of type 1 diabetes mellitus^35^. Our study provides some unique insight into the HLA loci associated with type 1 diabetes mellitus in the Arab and Asian populations. A study conducted in Bahrain, focused on HLA class II alleles found that HLA -DRB1*03:01:01 and -DQB1*02:01 is the most common in Bahrainis^36^. Another study from Kuwait reported a higher prevalence of HLA‐DQB1 non‐Asp57 allele *02:01, DQA1 Arg52 allele *03:01, and HLA‐DQA1*03:01/DQB1*02:01 alleles in their cohort of children with type 1 diabetes mellitus^37^. A similar study from Egypt on children with diabetes reported that 77.2% of these children had HLA-DR3-DQ2 haplotype^38^.

In this study we found that HLA -DQA1*03:01:01G (OR = 2.46, *p* value = 0.011) and -DQB1*03:02:01G (OR = 2.43, *p* value = 0.022) are enriched in Qatari children with type 1 diabetes mellitus. These alleles have been previously reported in the Caucasian population where they are in strong linkage disequilibrium with HLA-DRB1*4 and are an important risk factor to type 1 diabetes mellitus^13^. Susceptible HLA alleles are relatively common in the general population, but the key to the development of the disease is the combination of the HLA haplotypes inherited from parents^35,39^. In the Qatari population the association of HLA-DQB1*03 with HLA-DRB1*04 subtypes appears to be very poor. The disturbed association might be the result of recent admixture in the Qatari populations^40^. The enrichment of HLA-DQB1 and not HLA-DR, in our paediatric population might also be age-driven. The number of patients with only HLA-DQ, non-associated with HLA-DRB1*03/DRB1*04 increases with age, indicating a more indolent disease progression to reach clinical pathology^41,42^. In Qatar, type 1 diabetes mellitus is one of the most prevalent diseases in the paediatric setup, suggesting that HLA-DQ can be considered a major risk factor and disease predictor at an early age. In our study, HLA -DQA1*01:02:01G (OR = 0.17, *p* value = 3.4E−03) and -DQB1*06:02:01G (OR = 0, *p* value = 0.014) present protection against the disease in Qatari patients. The negative correlation of these alleles with type 1 diabetes mellitus has been reported in multiple ethnic groups and therefore, indicates a significant beneficial effect for the patients, even if they are carried as heterozygous with a risk allele^43^. During the autoimmune process, pancreatic beta-cells are destroyed by CD8^+^ T lymphocytes. CD8^+^T cells through their receptors (TCR) bind to HLA class I molecules presenting the antigenic peptide from the pancreatic islets. Thus, it is conceivable to consider the involvement of class I molecules in the susceptibility to disease. However, in this study the association of class I alleless with type 1 diabetes mellitus in Qatari patients was not detected.

The data presented here argues that specific HLA-class II alleles might be important players in susceptibility to type 1 diabetes mellitus in the Qatari population and can potentially be used as a biomarker of early onset of the disease. Both risk and protective alleles associated significantly with type 1 diabetes mellitus are different between Qatari and non-Qatari paediatric cohort, emphasizing the singularity of the Qatari population. Studying a larger cohort of paediatric patients is necessary to fully assess the contribution of HLA alleles in the susceptibility to and protection from type 1 diabetes mellitus. It will allow identification of the most important alleles that should be considered for inclusion in type 1 diabetes mellitus genetic screening in Qatar. Unraveling the type 1 diabetes mellitus risk factors in the Qatari population is an important step towards understanding the mechanism underlying the disease pathology and can potentially lead to the implementation of new intervention or prevention measures.

## Supplementary Information

Supplementary Information.

## Data Availability

The datasets generated during and/or analysed during the current study are not publicly available to protect patient confidentiality but are available from the corresponding author on reasonable request.
