# Supplementary material for: Clinical features, epidemiology, autoantibody status, HLA haplotypes and genetic mechanisms of type 1 diabetes mellitus among children in Qatar
Source: Sci Rep. 2021 Sep 23;11:18887. doi: 10.1038/s41598-021-98460-4 (PMC8460652; doi:10.1038/s41598-021-98460-4)
Supplement: Supplementary file 1 — Supplementary Information. [file 41598_2021_98460_MOESM1_ESM.docx]

**Supplementary Files**

**Clinical features, Epidemiology, Autoantibody status, HLA Haplotypes and Genetic Mechanisms of Type 1 diabetes mellitus among Children in Qatar**

Short title: Childhood Type 1 diabetes mellitus in Qatar: Clinical features, Autoantibody status, HLA haplotypes and genetic mechanisms.

Basma Haris^1^, Ikhlak Ahmed^2^, Najeeb Syed^2^, Hakeem Almabrazi^2^, Saras Saraswathi^1^, Sara Al-Khawaga^1^, Amira Saeed^1^, Shihab Mundekkadan^1^, Idris Mohamed^1^, Sanaa Sharari^1^, Iman Hawari^1^, Noor Hamed^1^, Houda Afyouni^1^, Tasneem Abdel-Karim^1^, Shayma Mohammed^1^, Amel Khalifa^1^, Maryam Al-Maadheed^1^, Mahmoud Zyoud^1^, Ahmed Shamekh^1^, Ahmed Elawwa^1^, Fawziya Al-Khalaf^1^, Zohreh Tatari-Calderone^2^, Goran Petrovski^1^, Khalid Hussain^1^*

1. Division of Endocrinology, Department of Paediatrics, Sidra Medicine, Doha, Qatar

2. Translational Research, Sidra Medicine, Doha-Qatar

Corresponding Author-

Professor Khalid Hussain MBChB MD MRCP MRCPCH MSc

Professor of Paediatrics, Weill Cornell Medicine-Qatar

Division Chief – Endocrinology, Department of Paediatric Medicine

Division of Endocrinology

Al Luqta Street, Education City North Campus,

PO Box 26999

Doha, Qatar

Direct +974-4003-7608 | MOB +974-30322007

[khussain@sidra.org](mailto:khussain@sidra.org)| [www.sidra.org](http://www.sidra.org/)

Honorary Professor University College London UK

Supplementary table 1- Genes associated with type 1 diabetes from previous studies

| Type 1 diabetes genes | | |
| --- | --- | --- |
| UBASH3A | IL2RA | CD226 |
| TYK2 | IL27 | CCR7 |
| TNFAIP3 | IL21 | CCR5 |
| TAGAP | IL2 | CENPW |
| STAT4 | IL18RAP | C1QTNF6 |
| SMARCE1 | IL10 | RNLS |
| SKAP2 | IKZF1 | BAD |
| SIRPG | IFIH1 | BACH2 |
| SH2B3 | GLIS3 | AFF3 |
| RGS1 | GAB3 |  |
| PTPN22 | FUT2 |  |
| PTPN2 | ERBB3 |  |
| PRKD2 | DLK1 |  |
| PRKCQ | CYP27B1 |  |
| ORMDL3 | CTSH |  |
| NRP1 | CTRB1 |  |
| CLEC16A | CTLA4 |  |
| ITGB7 | COBL |  |
| INS | CTRP6 |  |
| IL7R | CD69 |  |

Supplementary Table 2- Frequency spectrum of the significantly enriched alleles in Qatari patients and controls.

| Allele | Allele frequency (cases) | Allele frequency (controls) | p.value | odds-ratio | (95% CI) |
| --- | --- | --- | --- | --- | --- |
| DQA1*01:02:01G | 4% | 22% | 3.43E-03 | 0.17 | 0.03-0.63 |
| DQA1*03:01:01G | 41% | 17% | 1.19E-02 | 2.46 | 1.16-5.5 |
| DQB1*03:02:01G | 38% | 15% | 2.26E-02 | 2.43 | 1.11-5.6 |
| DQB1*03:02:01G | 0% | 9% | 1.40E-02 | 0.00 | 0-0.71 |

| Supplementary Table 3- Frequency spectrum of the significantly enriched alleles in non-Qatari patients and controls. |
| --- |
| \| Allele \| Allele frequency (cases) \| Allele frequency (controls) \| p.value \| odds-ratio \| (95% CI) \| \| --- \| --- \| --- \| --- \| --- \| --- \| \| A*03:01:01G \| 10% \| 1% \| 9.55E-03 \| 7.04 \| 1.29-71.33 \| \| B*08:01:01G \| 9% \| 1% \| 7.71E-03 \| 12.04 \| 1.42-562.37 \| \| B*49:01:01G \| 9% \| 1% \| 2.17E-02 \| 6.03 \| 1.04-62.71 \| \| C*07:01:01G \| 19% \| 7% \| 3.69E-02 \| 2.63 \| 1.01-7.07 \| \| DQB1*03:01:01G \| 1% \| 17% \| 1.92E-03 \| 0.09 \| 0-0.57 \| \| DRB1*03:01:01G \| 46% \| 18% \| 3.03E-03 \| 2.51 \| 1.32-4.81 \| \| DRB3*01:01:02G^*^ \| 18% \| 41% \| 1.42E-02 \| 0.43 \| 0.2-0.88 \| |

^*^Presence of HLA-DRB3:01:01:02 is due to LD with HLA-DRB1*03:01:01
